# Case Report: Anlotinib Reverses Nivolumab Resistance in Advanced Primary Pulmonary Lymphoepithelioma-Like Carcinoma With FGFR3 Gene Amplification

**DOI:** 10.3389/fonc.2021.749682

**Published:** 2021-10-08

**Authors:** Yanyang Liu, Lang Long, Jiewei Liu, Lingling Zhu, Feng Luo

**Affiliations:** Lung Cancer Center, Cancer Center, and State Key Laboratory of Biotherapy, West China Hospital of Sichuan University, Chengdu, China

**Keywords:** pulmonary lymphoepithelioma-like carcinoma, *FGFR3*, anlotinib, nivolumab, anti-PD-1 resistance

## Abstract

**Background:**

Primary pulmonary lymphoepithelioma-like carcinoma (LELC) is a rare type of non-small cell lung cancer (NSCLC). Currently, anti-programmed death-1 (PD-1)/programmed death ligand-1 (PD-L1) has become an important treatment for NSCLC. Anti-human PD-1 monoclonal antibodies, such as nivolumab, significantly prolong the survival time of patients with advanced lung adenocarcinoma and lung squamous cell carcinoma. However, there are few reports on the therapeutic effect, drug resistance mechanism, and strategies to overcome resistance to anti-PD-1/PD-L1 treatment in advanced pulmonary LELC. We report the case of a patient with advanced pulmonary LELC harboring fibroblast growth factor receptor (FGFR)3 gene amplification that showed resistance to nivolumab. After treatment with anlotinib, a multi-targeted small-molecule tyrosine kinase inhibitor, the patient’s resistance to nivolumab was reversed. She achieved long-term disease remission with a combination of anlotinib and nivolumab treatment.

**Case Presentation:**

A 68-year-old woman was diagnosed with stage IVA pulmonary LELC. After multiple-line chemotherapy, her disease progressed. Since the PD-L1 expression rate of the patient was 90%, nivolumab was administered. However, the therapeutic effect of nivolumab was not ideal; the disease continued to progress, and a new cervical lymph node metastasis appeared. *FGFR3* gene amplification was detected in lymph node metastasis. Based on this gene abnormality, we added anlotinib to the treatment. After two cycles of anlotinib and nivolumab, the metastatic focus of the patient was significantly reduced. The patient continued to receive this combined treatment and achieved remission for more than 15 months.

**Conclusion:**

Pulmonary LELC with *FGFR3* gene amplification may not respond well to nivolumab monotherapy. The combination of anlotinib and nivolumab can reverse the resistance to nivolumab in pulmonary LELC with *FGFR3* gene amplification.

## Introduction

Non-small cell lung cancer (NSCLC) is a malignant tumor with the highest morbidity and mortality rates globally. Primary pulmonary lymphoepithelioma-like carcinoma (LELC) accounts for less than 1% of NSCLC cases (1). It is a rare subtype of NSCLC and is typically observed in young, non-smoking Asian populations. Early-stage pulmonary LELC is treated mainly by surgery, and the advanced stage is usually treated with comprehensive therapies based on chemotherapy and radiotherapy. Although patients with pulmonary LELC have better prognosis than those with other subtypes of NSCLC, some patients still show resistance to therapy and present with local tumor recurrence and/or distant tumor metastasis early (2, 3). There is no definitive cure for patients with advanced pulmonary LELC after failure of chemoradiotherapy, emphasizing the urgency to identify new drugs.

Immune checkpoint blockade therapy has profoundly changed the treatment paradigm in advanced NSCLC by improving outcomes. Anti-programmed death-1 (PD-1)/programmed death ligand-1 (PD-L1) monoclonal antibodies, such as nivolumab or pembrolizumab, significantly prolong the survival of patients with advanced NSCLC. However, most data on anti-PD-1/PD-L1 treatment for NSCLC are from lung adenocarcinoma and lung squamous cell carcinoma, and the data on anti-PD-1/PD-L1 on pulmonary LELC are limited. Although a few studies have reported the effect of anti-PD-1 monoclonal antibody on pulmonary LELC, the results are contradictory (4–6). Moreover, the resistance mechanisms and approaches to reverse the resistance of anti-PD-1 in pulmonary LELC are yet to be elucidated.

The fibroblast growth factor receptor (FGFR) pathway controls many cellular biological processes, such as cell cycle progression, metabolism, proliferation, survival, differentiation, and migration. FGFRs constitute a family of four tyrosine kinase receptors, from FGFR1 to FGFR4. Molecular aberrations in the FGFR and FGFR signaling pathways have been found in various solid malignant tumors, including bladder cancer, breast cancer, and NSCLC (7). Aberrant activation of FGFR signaling is central to angiogenesis, embryogenesis, inflammation, and cell proliferation in many cancers. It also suggests that FGFR plays a key role in signal transduction in lung cancer. Gene mutations, gene amplification, and chromosomal translocation of the FGFR family have been found in NSCLC and small cell lung cancer (8). Preclinical studies have indicated that FGFR inhibitors can inhibit growth and induce apoptosis in lung cancer cells harboring abnormal FGFR expression (9). Anlotinib is a newly developed oral multi-targeted receptor tyrosine kinase (RTK) inhibitor. It was originally designed to inhibit vascular endothelial growth factor receptor (VEGFR)1/2/3, FGFR1/2/3/4, platelet-derived growth factor-α (PDGFRα), and c-Kit with high affinity. Clinical trials have indicated that anlotinib significantly prolonged progression-free survival and overall survival of patients with advanced NSCLC (10). Anlotinib has been approved as a third-line treatment for NSCLC in China. Currently, novel evidence indicates that anlotinib and other FGFR inhibitors can enhance PD-1 checkpoint blockade therapy (11, 12). This brings a new perspective to the exploration of the role of FGFR in cancer immune therapy.

Here, we report a case of anlotinib that successfully reversed resistance to nivolumab in a female patient with advanced primary pulmonary LELC harboring *FGFR3* gene amplification.

## Case Description

**Figure 1:**
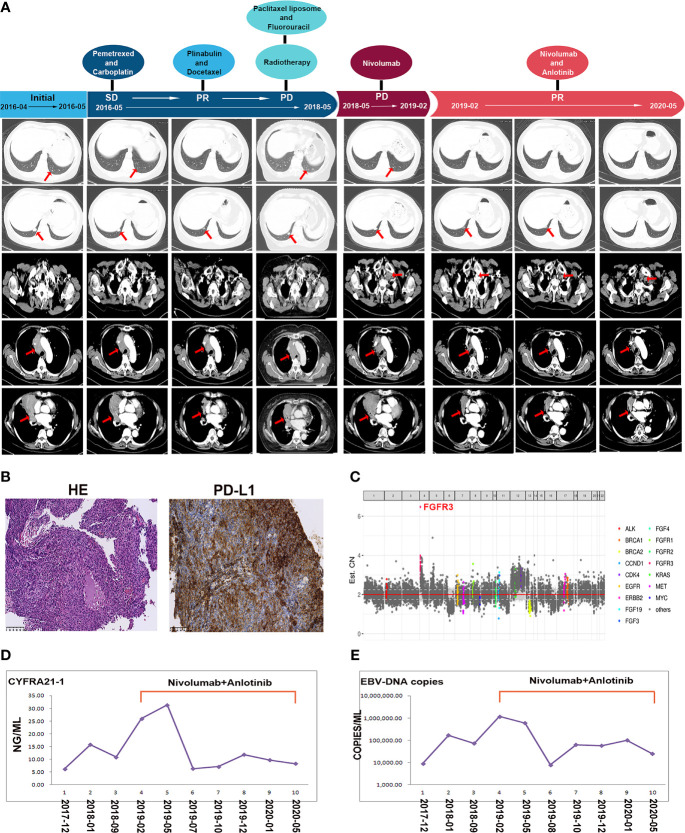
**(A)** Schematics show the treatment history of the patient. Enhanced computed tomography scan reveals the clinical response to chemotherapy, radiotherapy, nivolumab alone, and nivolumab combined with anlotinib. Red arrows point to the masses in pulmonary and lymph node metastases. **(B)** Immunohistochemistry presents the programmed death ligand-1 expression of the lymphoepithelioma-like carcinoma (LELC) tissues in pulmonary LELC. **(C)** The genetic testing result of the new metastatic lymph node in our patient, when her disease progressed after nivolumab monotherapy. **(D)** The curves showed serum tumor marker CYFRA21-1 levels before and after the combined nivolumab and anlotinib therapy. **(E)** The curves showed Epstein–Barr virus copy numbers in the serum before and after the combined nivolumab and anlotinib therapy.

A 68-year-old woman visited the hospital because of cough and sputum for 1 year in 2016. She was diagnosed with pulmonary LELC with mediastinal lymph node and bilateral pulmonary metastases. Immunohistochemical staining demonstrated the cancer cells labeled with pan-cytokeratin (+), P63 (+), cytokeratin (CK) 5/6 (+), epithelial membrane antigen (+), CK7 (−), thyroid transcription factor-1 (−), leukocyte common antigen (−), and Epstein–Barr virus (EBV)-encoded small RNA 1/2-ISH (+). She was administered 14 cycles of chemotherapy (first-line: pemetrexed and carboplatin; second-line: docetaxel and plinabulin; third-line: liposomal paclitaxel and fluorouracil) and underwent intensity-modulated radiation therapy (50.4 Gy/28F) of the chest from 2016 to 2018. This was marked by disease progression. As the PD-L1 proportion score of her primary lesion was 90%, she was administered nivolumab (200 mg every 2 weeks) alone in May 2018. The effect was not optimal as the lesion continued to grow. In February 2019, the pulmonary lesions and the left lymph node enlarged. An excisional biopsy of the neck lymph node revealed metastases with a PD-L1 proportion score of 5% and 1.6 mutations/Mb. *FGFR3* gene amplification was detected in the metastatic lymph nodes by next-generation sequencing (NGS). She was then administered a combination therapy of nivolumab (200 mg every 2 weeks) plus anlotinib (12 mg once daily on days 1–14 of each 21-day cycle). After nivolumab and anlotinib therapy for 3 months, a significant decrease in the size of the neck lymph node and lung mass was observed, and the EBV copy number in the serum, which was associated with a poor prognosis of LELC, dramatically decreased. She tolerated this combination treatment well, with a mild increase in blood pressure. Regular examination suggested that nivolumab combined with anlotinib was effective until May 2020 (**Figure 1**).

## Discussion

LELC mainly occurs in the foregut-derived organs, including the nasopharynx, salivary gland, thymus, stomach, and lung, and the 2015 World Health Organization classification scheme categorizes LELC under other and unclassified carcinomas (13). Primary pulmonary LELC was first described in a Southeast Asian woman by Bégin et al. (14) in 1987. It is a rare lung cancer, occurring in only 0.92% of patients, and is considered to be related to EBV infection. Because of the rarity of pulmonary LELC, the treatment experience is limited. Pulmonary LELC therapy is often based on the treatment of lung squamous cell carcinoma, and comprehensive therapies, such as surgery, chemotherapy, and radiotherapy, are all applied (1). Although previous studies have indicated that the prognosis of pulmonary LELC is better than that of other NSCLCs, some patients do not respond well to chemoradiotherapy. Advanced pulmonary LELCs that progress after chemotherapy and radiotherapy lack standard treatment; thus, there is a need to explore novel treatment strategies for this condition. Molecular targeted therapy is another important treatment for NSCLC. However, epidermal growth factor receptor (EGFR) gene mutations or anaplastic lymphoma kinase (ALK) gene rearrangements are rare in pulmonary LELC. Liu et al. (15) have investigated 32 patients with pulmonary LELC, but no exon 19 del or exon 21 L858R of *EGFR* mutation was detected. Chang et al. (16) have reported an approximately 17.39% (8/46) of patients with pulmonary LELC with *EGFR* mutations but without classical *EGFR* mutation sites such as L858R in exon 21 (16). *ALK* gene rearrangement is rarely observed in pulmonary LELC. Wang et al. (17) have detected a cohort of 42 patients with primary pulmonary LELC, genotyped for *ALK* rearrangement and *EGFR* mutation. None of the 42 patients had *ALK* rearrangement, and only one (2.4%) patient harbored the L858R mutation, but objective response to gefitinib was not observed (17). These results suggest that *EGFR* or *ALK* gene-targeted therapy is not a recommended treatment for advanced pulmonary LELC.

Currently, immune checkpoint inhibitors (ICIs), represented by anti-PD-1, anti-PD-L1, and anti-CTLA-4 antibodies, have radically changed the landscape of lung cancer treatment, demonstrating durable responses and possible treatment in some patients with advanced NSCLC (18). PD-L1 positivity is associated with the response rate to anti-PD-1/PD-L1 therapy. The anti-PD-1 antibody response rates are higher in cancers with elevated PD-L1 expression (19). Compared with the low rate of *EGFR* and *ALK* gene mutation, the positive rate of PD-L1 expression in pulmonary LELC is high. Wu et al. (20) found that 96.61% of patients with pulmonary LELC (57/59) have PD-L1 expression ≥1%, and 61.01% of patients with pulmonary LELC (36/59) have PD-L1 expression ≥50%. Fang et al. (21) have analyzed PD-L1 expression in 113 patients with pulmonary LELC who were surgically treated. The overall incidence of PD-L1 overexpression was 74.3% (84/113). High PD-L1 expression was associated with lower disease-free survival compared to low PD-L1 expression (21). This evidence suggests that pulmonary LELC might be a good candidate for ICI therapy. In recent years, seven case reports and two retrospective analyses have reported the efficacy of ICI in advanced pulmonary LELC (2, 22). The majority of these studies have reported a benefit from ICI therapy in pulmonary LELC. These data indicate that anti-PD-1 therapy may be an appropriate treatment for patients with advanced pulmonary LELC. However, the treatment effects of anti-PD-1 therapy in different patients with pulmonary LELC reported in the literature were significantly heterogeneous. Some patients with pulmonary LELC have a PD-L1 score higher than 50%, but their responses to anti-PD-1 therapy were stable disease. Although other patients had a PD-L1 score of only approximately 10%, they achieved a partial response (PR). Similarly, although the PD-L1 proportion score of our patient in this case was 90%, her response to nivolumab was limited, and she eventually developed a new lymph node metastasis during nivolumab treatment. The heterogeneity response suggests that factors other than PD-L1 expression levels might affect the response to ICI in pulmonary LELC. This issue has not yet been fully discussed in the available literature, and further studies are needed to explore this issue.

When the disease in our patient progressed after nivolumab treatment, we detected a new lymph node metastasis by NGS and found *FGFR3* gene amplification. *FGFR3* gene is located on chromosome 4p16.3. Its protein product, *FGFR3*, is a member of the FGFR family. Activating mutations of *FGFR3* are relatively common in bladder and uterine carcinomas and are associated with cancer cell proliferation and migration (23). However, *FGFR3* gene alterations are rarely observed in patients with NSCLC. *FGFR3* hotspot mutations, R248C and S249C, and FGFR3 fusions are found in 0.1% and 0.14% of NSCLC cases, respectively (24). In pulmonary LELC, the actionable alteration *FGFR3* (FGFR3 R248C and *FGFR3-TACC3* fusion) was 3.39% (2/59), as detected by Chau et al. (25). *FGFR3* gene amplification in pulmonary LELC was first reported in our case. The role of aberrant FGFR3 in pulmonary LELC is currently unclear. Notably, novel evidence indicates that FGFR has been established as a key modulator of the immune microenvironment in several cancers. FGFR3 pathway aberration and elevated FGFR1 expression and *FGFR2* gene mutation were associated with cancers that exhibit a lymphocyte-excluded phenotype (26, 27). Besides tumor cell intrinsic factors (such as negative PD-L1 expression, low tumor mutational burden), a noninflamed tumor microenvironment (TME) with limited infiltrating T cells is another important mechanism leading to ICI resistance (28). And in pulmonary LELC, CD8+ T cells play a contributing role in anticancer immunity (29). Thus, theoretically, the aberrant activation of the *FGFR* gene/FGFR pathway may influence response to ICI by rendering a non-T cell-inflamed TME in pulmonary LELC. A preclinical study has demonstrated that FGFR inhibition sensitizes the effect of PD-1 blockade by facilitating T-cell infiltration in lung cancer models (11). Based on these results, we expected to improve the response to nivolumab in our patient by FGFR inhibition.

Anlotinib, an RTK inhibitor targeting VEGFR1/2/3, FGFR1/2/3/4, c-Kit, and PDGFRα, has been approved by the China National Medical Products Administration for patients with advanced NSCLC who have progressed on at least two lines of prior systemic chemotherapy (10). Since other FGFR inhibitors, such as erdafitinib or rogaratinib, are not approved for the treatment of lung cancer in China, anlotinib was recommended to the patient in our case. Anlotinib combined with nivolumab yielded favorable results, and the patient achieved a PR for more than 15 months. Similarly, two recent studies with small sample sizes have reported that the combination of anlotinib and anti-PD-1 drugs has promising efficacy and manageable toxic effects in patients with advanced lung adenocarcinoma and lung squamous cell carcinoma (12, 30). The potential explanation for this synergistic effect is anlotinib may improve the ratio of CD8+ T cells/Foxp3+ cells in tumors by suppressing VEGFR-AKT signaling and thus improve immunotherapy effectiveness (31). Beyond this, we consider that there may be a second situation in our case, that is, pulmonary LELC developed an enhanced FGFR3 pathway, which could lead to lymphocytic exclusion leading to nivolumab resistance, and anlotinib resensitized the response to nivolumab in the patient by targeting FGFR3. However, we have only one case, and further studies should be conducted to verify this hypothesis.

In conclusion, this is the first report showing that anlotinib reversed the resistance to nivolumab in advanced pulmonary LELC with *FGFR3* gene amplification. This case indicates that *FGFR3* gene amplification is an adverse factor for nivolumab monotherapy in pulmonary LELC. The combination of nivolumab and anlotinib may be a new potential therapeutic strategy for improving the ICI effect in patients with pulmonary LELC with *FGFR3* gene aberration.

## Data Availability Statement

The datasets for this article are not publicly available due to concerns regarding participant/patient anonymity. Requests to access the datasets should be directed to the corresponding author.

## Ethics Statement

The studies involving human participants were reviewed and approved by the ethics committee of West China hospital. The patient/participant provided her written informed consent to participate in this study. Written informed consent was obtained from the individual(s) for the publication of any potentially identifiable images or data included in this article.

## Author Contributions

YL and LL collected the clinical information, diagnostic information, therapeutic information, and images of the patients. YL wrote the article. YL and LL identified the case and submitted the article. JL, LZ, and FL revised the article. JL proofread the article. YL and LL have contributed equally to this work. All authors contributed to the article and approved the submitted version.

## Funding

This work was supported by grants from National Natural Science Foundation of China (grant no. 82003089).

## Conflict of Interest

The authors declare that the research was conducted in the absence of any commercial or financial relationships that could be construed as a potential conflict of interest.

## Publisher’s Note

All claims expressed in this article are solely those of the authors and do not necessarily represent those of their affiliated organizations, or those of the publisher, the editors and the reviewers. Any product that may be evaluated in this article, or claim that may be made by its manufacturer, is not guaranteed or endorsed by the publisher.
